# Supplementary material for: The Genomic and Genetic Evolution Analysis of Rabbit Astrovirus
Source: Vet Sci. 2022 Oct 31;9(11):603. doi: 10.3390/vetsci9110603 (PMC9697364; doi:10.3390/vetsci9110603)
Supplement: Supplementary file 1 [file vetsci-09-00603-s001.zip › vetsci-1956853-supplementary.pdf]

Supplementary Table S1. List of the 37 sequences used in this study.

GenBank accession number	Strain name	Host	Year of isolation	Location
KC285152	HAstV-2/RUS/Novosibirsk/Nsc11-N2411/2011	Homo sapiens	2011	Russia
KF039911	Rus-Nsc06-1029	Human	2006	Russia
L13745	Oxford	Human	1993	USA
KF039910	Rus-Nsc05-430	Human	2005	Russia
AF141381	Berlin	Human	1999	Germany
L23513	Oxford	Human	1994	USA
MF684776	2013/Fuzhou/85	Homo sapiens	2013	China
KF039913	Rus-Nsc10-N358	Human	2010	Russia
KF039912	Rus-Nsc05-623	Human	2005	Russia
KP862744	kor85	Human	2014	South Korea
AF260508	Yuc-8	Human	2000	Mexico
NC_025379	PAstV-GX1	swine	2013	China
KF499111	1637F	Felis catus	2012	China
JN420359	9822	sea lion	2010	USA
FJ890352	CSL2	California sea lion	2008	USA
NC_025346	TN/2208/2010	Oryctolagus cuniculus	2010	USA
JN052023	rabbit/Nausica/2008/ITA	Rabbit	2008	Italy
**MZ682112**	**Z317**	**Rabbit**	**2020**	**China**
HM447045	CcAstV-1	Capreolus capreolus	2010	Denmark
JX556690	AstV2-US-IA122	swine	2011	USA
MK987102	BoAstV-VC65/693	Bos taurus	2016	Switzerland
JX556692	AstV4-US-IL135	swine	2011	USA
MK987099	BoAstV-VC34/338	Bos taurus	2016	Switzerland
MK613068	PAstV/CH/HB-SJZ/2018	porcine	2018	China
MW249010	DesRot/Peru/AYA14_F_DrAstV	Desmodus rotundus	2016	Peru
MT734809	BAstV/RB	Bat	2020	USA
NC_019494	US-MO123	swine	2011	USA
KF859964	BF34	Homo sapiens	2010	Burkina Faso
MN464146		Bos taurus breed Marchigiana	2019	Italy
LC341267	BoAstV/JPN/KagoshimaSR28-462/2016	Bos taurus	2016	Japan

MK987103	BoAstV-VC65/698	Bos taurus	2016	Switzerland
MK987100	BoAstV-VC34/346	Bos taurus	2016	Switzerland
NC_023636	AstV5-US-IA122	swine	2011	USA
MT585643	VF14-92-A2	Gallus gallus broiler chicken	2020	United Kingdom
JF414802	GA2011	chicken	2007	USA
EU143850	TAstV/TX/00	Turkey	2007	USA
FJ434664	C-NGB	Duck	2008	China
